# Predicting walking capacity, walking performance, and life space mobility using socket comfort in lower-limb prosthesis users

**DOI:** 10.1177/02692155251374927

**Published:** 2025-09-15

**Authors:** Gordon Tao, Jayden Singh, Malena Rapaport, Michael Payne, William C Miller

**Affiliations:** 1 Rehabilitation Research Program, Centre for Aging SMART at Vancouver Coastal Health, Vancouver, BC, Canada; 2 Department of Occupational Science and Occupational Therapy, 8166 The University of British Columbia, Vancouver, BC, Canada; 3 Graduate Program in Rehabilitation Sciences, Faculty of Medicine, 8166 The University of British Columbia, Vancouver, BC, Canada; 4 School of Kinesiology, Faculty of Education, 141631 The University of British Columbia, Vancouver, BC, Canada; 5 Prosthetics & Orthotics Program, 2098 British Columbia Institute of Technology, Burnaby, British Columbia, Canada; 6 Department of Physical Medicine & Rehabilitation, 70384 Schulich School of Medicine & Dentistry, University of Western Ontario, London, ON, Canada

**Keywords:** Socket comfort, walking capacity, walking performance, life space mobility

## Abstract

**Objective:**

To determine if socket comfort predicts walking capacity, walking performance, and life-space mobility.

**Design:**

Observational exploratory study involving a secondary cross-sectional analysis using multiple regression of data collected from an exercise intervention trial.

**Setting:**

Laboratory setting for clinical assessments; community setting for walking performance.

**Participants:**

Community-dwelling lower-limb prosthesis users over 50 years old (*n* = 72).

**Main measures:**

Socket Comfort Score, 2-Minute Walk Test, step count, Life Space Assessment, and control variables including demographics, Short Physical Performance Battery, Four Square Step Test, and Walking While Talking.

**Results:**

Regression modeling showed Socket Comfort Score as a statistically significant predictor of 2-Minute Walk Test (*B* = 6.9 m, 95% CI [2.7, 11.1] m) alongside amputation level, Walking While Talking test, and Short Physical Performance Battery (greatest contribution to the model); the model accounted for 61% of the variance. Socket Comfort Score was not a statistically significant predictor of step count. Socket Comfort Score was the only statistically significant predictor of Life Space Assessment (*B* = 4.9, 95% CI [1.1, 8.8]); the model accounted for 12% of the variance.

**Conclusions:**

Socket comfort played a notable role in predicting walking capacity and life space mobility, but not in walking performance. Improving lower extremity function may have greater impact on walking overall. While this study provides context regarding socket comfort that clinicians may consider when planning holistic prosthetic rehabilitation, mixed findings in the literature suggest that further research on how socket comfort relates to walking outcomes in the community is warranted.

## Introduction

Lower limb amputation reduces physical ability and can negatively affect quality of life, including increased chronic pain, reduced mental well-being, and reduced community participation.^1,2^ Addressing these impacts, a lower limb prosthesis is commonly used to restore walking capacity and support participation.^3^ Improved walking capacity (amount one can walk) is well correlated with walking performance (amount one does walk).^4^ Accordingly, lower limb prosthesis users often describe their community mobility in terms of barriers and facilitators of walking performance,^5^ which can be conceptualized as life space mobility (the extent to which an individual independently moves through their community), which reflects participation.^6^ While some lower limb prosthesis users regain high-level physical activity,^7^ many still experience challenges in these walking-related outcomes, for example, pain, fatigue, or environmental obstacles like winter conditions.^8^ The worst of these challenges can lead to rejection of the prosthesis.^9^ Therefore, improving walking-related remains an ongoing priority for engaging lower limb prosthesis users in community activity.

One important determinant of walking in lower limb prosthesis users is socket comfort. In clinical practice, addressing socket comfort and pain are primary goals of prosthetic rehabilitation, as patients (both new and experienced) are more likely to use their prosthesis if it is comfortable. Socket comfort is multifactorial, influenced by amputation etiology, residual limb characteristics (e.g. length, shape, edema, atrophy, bony contours, and nerve endings), as well as the interface and loading surfaces of the prosthesis.^10^ Pain and discomfort are a central concerns of many lower limb prosthesis users,^11^ with socket comfort or pain being associated with reduced perceived mobility^12^ and cited in prosthesis rejection, leading to reduced participation in activities requiring prosthesis use.^13^ While socket comfort clearly impacts walking outcomes, its specific role among other determinants of walking remains underexplored.

Beyond socket comfort, personal factors, pre-amputation lifestyle, amputation level, physical function, balance, and cognitive ability all influence walking capacity, walking performance, and community participation in lower limb prosthesis users.^14^ This complexity makes it difficult for clinicians to situate the role of socket comfort and anticipate its impact on walking performance and life space mobility once patients leave the clinic. Clarifying its predictive role could help improve socket evaluations, guide prosthetic rehabilitation, and mitigate prosthesis rejection. Therefore, the objective of this study was to determine the extent to which socket comfort is associated with walking capacity, walking performance, and life space mobility among lower limb prosthesis users among other key factors.

## Methods

This was an observational exploratory study and a secondary cross-sectional analysis of baseline data collected from an exercise intervention study (parent study) for persons with lower limb amputation.^15^ The parent study received ethical approval from University of British Columbia Clinical Research Ethics Board (Approval #: H13-01858), University of Western Ontario Health Sciences Research Ethics Board (Approval #: 104688), and University of Alberta Health Research Ethics Board (Approval #: Pro00061142).

Data collected during the parent study from 72 participants were analyzed in this study. The parent study took place in Vancouver, BC, London, ON, and Edmonton, AB, and required participants to be community-dwelling adults over 50 years of age with a unilateral transtibial or transfemoral amputation. They needed to have used their prosthesis for at least 2 hours/day for the past 6 months and be cognitively able to engage in the program (modified mini-mental status exam score above 24). The parent study excluded participants if they could not provide informed consent in English, had medical conditions contraindicating exercise participation,^16^ had prosthesis fit issues (Socket Comfort Score < 6), or were participating in another exercise program or rehabilitation study. All participants provided informed consent for the parent study.

After screening, demographics (age, sex, amputation level, months since amputation) were collected and clinical measures were assessed in a laboratory setting. These data were collected in one session for each participant. Data for walking performance were collected during the following week.

Socket comfort was measured using the Socket Comfort Score,^17^ which is a single item self-report measure rated from 0 (lowest comfort) to 10 (highest comfort). It has demonstrated test reliability with an intraclass correlation coefficient between 0.63 and 0.37.^17^

Walking capacity was measured using the 2-Minute Walk Test,^18^ which captures meters walked over a flat surface indoors over two minutes. It has demonstrated high intra and inter-rater reliability (intraclass correlation coefficients of .90–.96 and .98–.99, respectively),^18^ as well as responsiveness to change in lower-limb amputees.^18^

Walking performance was measured using step count. During their assessment session, participants were provided and instructed to wear the Modus Health Stepwatch Activity Monitor (Modus Health, Mountlake Terrace, USA) attached to their prosthesis ankle over the following week. Step count was recorded at the end of the week. The Stepwatch Activity Monitor has been shown to be accurate in community-dwelling populations^19^ and persons with irregular walking patterns.^20^

Life space mobility was measured using the Life Space Assessment, which is a self-report measure that captures the breadth and frequency of mobilization within five life-space levels, from within one's home to outside of town, with or without use of mobility aids.^21^ The Life Space Assessment is scored from 0 (totally bed-bound) to 120 (traveling out of town every day without assistance), and has demonstrated test-retest reliability with an intraclass correlation coefficient of .86.^21^

As this secondary analysis involved a pre-determined sample and set of measures, control variables were selected based on the authors’ clinical experience and biological plausibility as well as informed by the International Society for Prosthetics and Orthotics’ minimum data set.^22^ We selected measures to control for lower limb function, dynamic balance, and cognitive-motor ability. Demographic control variables included age, sex, amputation level, and time since amputation.

Lower extremity function was measured using the Short Physical Performance Battery, which was modified for lower limb prosthesis users in the parent study.^15^ Our modified battery involved 5 tasks scored by a clinician from 0 to 6 for a total score from 0 to 30, with a higher score indicating greater function. The unmodified Short Physical Performance Battery has demonstrated high test-retest reliability (intraclass correlation coefficient = .92).^23^

Dynamic balance was measured using the Four Square Step Test,^24^ which involves stepping through a 2 × 2 grid on the floor in clockwise and counter-clockwise direction while being timed, with shorter times indicating better dynamic balance. The Four Square Step Test has demonstrated high reliability (intraclass correlation coefficient = .98).^24^

Cognitive-motor ability was measured using the Walking While Talking test,^25^ which involves walking for 40 ft while reciting the alphabet with all letters (simple) and alternating letters (complex). Greater time difference between simple and complex indicates poorer ability. The Walking While Talking test has demonstrated moderate inter-rater reliability (*r* = .60).^25^

The sample was summarized using descriptive statistics of mean, standard deviation, and range. To examine the role of Socket Comfort Score in predicting 2-Minute Walk Test, Step Count, and Life Space Assessment we first used correlation to examine the bivariate relationship between socket comfort and clinical outcomes. This was followed by a sequence of multiple regression models including a demographic block (age, sex, amputation level, and months since amputation) followed by an additional clinical measures block (Short Physical Performance Battery, Four Square Step Test, and Walking While Talking test) for control variables. All statistical analyses were conducted in SPSS Statistics 25 (IBM, Armonk, USA) using an *α* of 0.05. Multicollinearity was checked using a variance inflation factor threshold of 2.5.

## Results

In total, 23 participants were recruited from Vancouver, BC, 36 from London, ON, and 13 from Edmonton, AB. Participants’ ages ranged from 51 to 89 (*M* = 65.3, SD = 8.8) years, with 10 participants identifying as female and 62 as male. Time since amputation ranged from 0.75 to 75 (*M* = 15.8, SD = 19.8) years, with 58 amputations below the knee and 14 at the knee or above. Complete data were collected for clinical measures Socket Comfort Score, Short Physical Performance Battery, 2-Minute Walk Test, and Life Space Assessment (Table 1). One participant skipped the Four Square Step Test, and another the Walking While Talking test due to discomfort on the assessment day. Step count data was missing from 5 participants due to equipment failure. These data were treated as missing at random and excluded from the analysis.

**Table 1.** Summary of clinical measures and step count.

	*n*	Mean (SD)	Range
SCS	72	8.1 (1.4)	6–10
2MWT (m)	72	125 (38)	31–218
SAM (steps)	67	2321 (1463)	86–6182
LSA	72	75 (23)	36–120
SPPB	72	15.0 (6.7)	3–28
FSST (s)	71	14.1 (7.3)	6.3–51.1
WWT Δ (s)	71	4.1 (7.8)	−2.3–53.9

Bivariate correlation showed that Socket Comfort Score was weakly^26^ and positively correlated with 2-Minute Walk Test, step count, and Life Space Assessment (Pearson correlation coefficient of 0.29, 0.24, and 0.24 respectively; *p* < .05 for all three).

Multiple regression models were completed, regressing each outcome on Socket Comfort Score, first controlling for the demographic variables only and then controlling for both demographic and clinical variables as well (Table 2). Small multicollinearity (2.5 ≤ variance inflation factor < 2.6) appeared between SPBB and Four Square Step Test for all three outcomes. Removing either did not meaningfully affect Socket Comfort Score's influence in the models. Therefore, we retained the control variable with the greater relative contribution and removed the other. All models are presented in Supplemental A.

**Table 2.** Multiple regression models for 2-minute walk test, SAM step count, and life space assessment.

2-Minute Walk Test ( $R_{\mathrm{adj}}^{2}=0.61$ )							
	Unstandardized		Standardized			95.0% CI for *B*	
	*B*	Std. Error	Beta	*t*	Sig.	Lower	Upper
(Constant)	32	30		1.04	0.30	−29	93
Age	−0.08	0.37	−0.02	−0.23	0.82	−0.83	0.66
Sex	−1.83	8.59	−0.02	−0.21	0.83	−18.99	15.34
Amp. Level	**−14**.**70**	**5**.**32**	**−0**.**21**	**−2**.**76**	**0**.**01**^a^	**−25**.**33**	**−4**.**07**
Months Since Amp.	0.01	0.01	0.05	0.57	0.57	−0.02	0.03
SCS	**6**.**87**	**2**.**11**	**0**.**25**	**3**.**26**	**0**.**00**^a^	**2**.**65**	**11**.**08**
SPPB	**3**.**43**	**0**.**53**	**0**.**60**	**6**.**49**	**0**.**00**^a^	**2**.**37**	**4**.**49**
WWT	**−1**.**66**	**0**.**37**	**−0**.**34**	**−4**.**53**	**0**.**00**^a^	**−2**.**39**	**−0**.**93**

**Table:** 

SAM Step Count ( $R_{\mathrm{adj}}^{2}=0.38$ )							
	Unstandardized		Standardized			95.0% CI for *B*	
	*B*	Std. Error	Beta	*t*	Sig.	Lower	Upper
(Constant)	−970	1512		−0.64	0.52	−3996	2057
Age	16.61	19.00	0.10	0.87	0.39	−21.42	54.64
Sex	**1196**	**440**	**0**.**28**	**2**.**72**	**0**.**01**^a^	**316**	**2076**
Amp. Level	**−804**	**285**	**−0**.**29**	**−2**.**82**	**0**.**01**^a^	**−1375**	**−233**
Months Since Amp.	0.88	0.73	0.14	1.21	0.23	−0.57	2.34
SCS	95	110	0.09	0.87	0.39	−124	315
SPPB	**85**	**27**	**0**.**38**	**3**.**16**	**0**.**00**^a^	**31**	**138**
WWT	2	19	0.01	0.10	0.92	−36	40

**Table:** 

Life Space Assessment ( $R_{\mathrm{adj}}^{2}=0.12$ )							
	Unstandardized		Standardized			95.0% CI for *B*	
	*B*	Std. Error	Beta	*t*	Sig.	Lower	Upper
(Constant)	57	24		2.36	0.02	9	104
Age	−0.16	0.32	−0.06	−0.49	0.63	−0.80	0.49
Sex	−4.40	7.76	−0.07	−0.57	0.57	−20	11
Amp. Level	−3.29	4.94	−0.08	−0.67	0.51	−13.17	6.59
Months Since Amp.	0.01	0.01	0.12	0.99	0.33	−0.01	0.03
SCS	**4**.**93**	**1**.**91**	**0**.**30**	**2**.**57**	**0**.**01**^a^	**1**.**10**	**8**.**76**
FSST	−0.87	0.46	−0.28	−1.88	0.06	−1.79	0.05
WWT	−0.08	0.40	−0.03	−0.20	0.84	−0.88	0.72

a Indicates statistically significant *α* < 0.05; default sex is female; default amp. level is below-knee.

The 2-Minute Walk Test model, including Socket Comfort Score and demographic variables only, explained 22% of the variance ( $R_{\mathrm{adj}}^{2}=0.22$ , *p* < .01) and 61.2% of the variance when also accounting for clinical measures ( $R_{\mathrm{adj}}^{2}=0.61$ , *p* < .01). In the demographics only model, Socket Comfort Score was a statistically significant predictor of 2-Minute Walk Test, with a unit increase in Socket Comfort Score predicting an increase of 7.8 m walked, 95% CI [2.0, 13.6]. In the full model (Table 2), Socket Comfort Score was a statistically significant predictor of 2-Minute Walk Test score, with a unit increase in Socket Comfort Score predicting an increase of 6.9 m walked, 95% CI [2.7, 11.1]. Other statistically significant predictors included amputation level, Short Physical Performance Battery, and Walking While Talking test (*p* < .01). Short Physical Performance Battery showed the greatest contribution to explaining the variance in 2-Minute Walk Test, with a standardized coefficient of 0.60, compared to 0.25 of Socket Comfort Score. This translated to an increase of 3.43 m, 95% CI [2.4, 4.5], in 2-Minute Walk Test for every unit increase in Short Physical Performance Battery score.

For Stepwatch Activity Monitor step count, the model including Socket Comfort Score and demographic variables only, explained 31% of the variance ( $R_{\mathrm{adj}}^{2}=0.31$ , *p* < .01) and 38% of the variance when also including clinical measures ( $R_{\mathrm{adj}}^{2}=0.38$ , *p* < .01). However, Socket Comfort Score was not a statistically significant predictor of Stepwatch Activity Monitor step count in either of these models (*p* = .39). Sex, amputation level, and Short Physical Performance Battery were statistically significant predictors of Stepwatch Activity Monitor step count (*p* < .05) and contributed similarly to explaining the variance in Stepwatch Activity Monitor step count, with absolute standardized coefficients ranging from 0.28 to 0.38.

The model for Life Space Assessment including only Socket Comfort Score and demographics was not statistically significant ( $R_{\mathrm{adj}}^{2}=0.012$ , *p* = .33). When also including clinical measures, the model explained 12% of the variance ( $R_{\mathrm{adj}}^{2}=0.12$ , *p* = .039). Socket Comfort Score was the only statistically significant predictor in this model, with one unit increase in Socket Comfort Score predicting an increase of 4.93, 95% CI [1.10, 8.76], in Life Space Assessment score.

## Discussion

This study analyzed the relationship between socket comfort and walking capacity, walking performance, and life space mobility. The initial correlation analyses showed statistically significant bivariate associations between socket comfort and walking capacity, walking performance, as well as life space mobility. While regression analyses showed that participants with higher socket comfort demonstrated greater walking capacity when accounting for demographics, dynamic balance and cognitive function, a similar association was not observed between socket comfort and walking performance, nor life space mobility.

These results confirm the notion that socket comfort can be a useful indicator of walking capacity but with limited granularity, as one unit increase of Socket Comfort Score was only associated with a 7-m increase in 2-Minute Walk Test, far below the 34-m minimal detectable change for lower limb prosthesis users.^27^ This suggests that meaningful improvements in walking capacity may require large improvements in socket comfort. Similarly, a study comparing different socket designs also found improved walking capacity with more comfortable sockets.^28^ Conversely, Pousett et al. found minimal correlation (.01 ≤ *r* ≤ .15) between socket comfort and walking capacity in both new and experienced prosthesis users.^12^ Moreover, lower extremity function (Short Physical Performance Battery) appeared to be the greater contributor to differences in walking capacity in our model. These findings suggest that improving socket comfort may be a secondary to improving lower limb function and complementary to improving cognitive ability for improving walking capacity. Still, given the typically low walking capacity of recent lower limb amputees,^18^ improving socket comfort could be an acute goal in prosthetic rehabilitation to help address their inactivity.^29^

After controlling for demographics and clinical measures, Socket Comfort Score was not a statistically significant predictor of step count, indicating socket comfort does not necessarily predict walking performance outside the clinic in prosthesis users similar to our sample. This contrasts with studies showing positive associations between socket comfort and prosthesis-related physical activity including step count.^1,30^ This discrepancy may be explained by our sample, which excluded lower limb prosthesis users with severe discomfort or pain (Socket Comfort Score < 6) and whose inclusion may have otherwise strengthened the association.^13^ It may also be that socket comfort influences walking performance only below a certain threshold, with little added benefit at higher comfort levels as seen in our sample. Additionally, very low step counts observed in some participants may have reflected periods of cosmetic rather than physical activity use. Nevertheless, other studies showing associations between socket comfort and activity in individuals with similarly high average comfort ratings^4,30^ as our sample suggests more work needs to be done to analyze this relationship.

In this study, lower extremity function, amputation level, and sex (each sharing similar explanatory power) were stronger predictors of walking performance that socket comfort. Below-knee amputees have been shown to walk more than above-knee amputees after amputation and in the years following,^31^ and our sample included a high proportion of the former. Additionally, our sample had more male participants than female; male lower limb prosthesis users tend to have better mobility.^32^ These findings underscore walking performance as a multifaceted outcome influenced by both personal and functional factors.

Socket comfort appeared to be a statistically significant predictor of life space mobility after controlling for demographics and clinical measures, with an effect size potentially exceeding the minimum clinically important change of 5 Life Space Assessment points when Socket Comfort Score improves by more than 1.^33^ Similarly, Pousett et al. found socket comfort to be positively correlated with perceived mobility during initial evaluation and definitive fitting for socket replacement.^12^ This suggests that socket comfort can influence one's perceptions of their ability to move through different life spaces. In contrast, Diment et al. showed that socket fit was not significantly correlated with community participation such as paid work or sport.^30^ This may reflect that long-distance mobility (such as between neighborhoods and cities) often involves vehicle transportation rather than walking; our sample included participants who travelled between cities regularly (high to maximum Life Space Assessment score). Ultimately, the low explanatory power of our model indicates that, while socket comfort is meaningful, other factors may play a larger role in explaining life space mobility and further research into its determinants among lower limb prosthesis users is required.

The study should be interpreted according to several limitations. As a secondary analysis, this study was primarily limited in generalizability by the sample of the parent study. Our sample included a high proportion of male and below-knee amputees who were relatively high-functioning, with high socket comfort scores, as those with serious prosthetic fit issues (SCS < 6) or medical conditions preventing exercise were excluded. Thus, our findings primarily reflect healthier, more comfortable lower limb prosthesis users. However, our sample's socket comfort scores align with those in other studies of community-dwelling users.^12^ While the Stepwatch Activity Monitor provides accurate step count, it only captures one dimension of walking performance. Other dimensions of walking performance such as the type of walking in the community (shuffling around the house or walking an energetic dog) may also relate to socket comfort.^7^ Finally, while this study assumed the frame of socket comfort as a modifiable factor (in clinic) that might influence changes in walking outcomes, this study was nevertheless a cross-sectional one and therefore does not address the longitudinal relationship between socket comfort and walking outcomes within individuals.

In conclusion, socket comfort and lower extremity function are both important factors related to walking outcomes in experienced or higher-functioning lower limb prosthesis users, when accounting for demographic and clinical factors. However, while improving socket comfort may help to improve mobility in clinical settings, it alone may not fully translate to activity in community environments. Lower extremity function may be more important for clinicians to focus on when planning care around improving walking performance in home and community environments. Overall, individuals with high socket comfort may require broader and more holistic interventions to see further improvement. While this study provides context regarding socket comfort that clinicians may consider when seeking to improve walking in lower limb prosthesis users, further research on the relationships between socket comfort, walking capacity, walking performance, and life space mobility is warranted.

**Clinical messages**

- Greater socket comfort was associated with greater walking capacity but not step count over a 1-week period.
- Improving lower extremity function is likely to be the priority for improving walking capacity and walking performance in the community over socket comfort.
- Greater socket comfort may help explain greater life space mobility to a small degree, but more research is needed on life space mobility overall.

## Supplemental Material

sj-docx-1-cre-10.1177_02692155251374927 - Supplemental material for Predicting walking capacity, walking performance, and life space mobility using socket comfort in lower-limb prosthesis users Supplemental material, sj-docx-1-cre-10.1177_02692155251374927 for Predicting walking capacity, walking performance, and life space mobility using socket comfort in lower-limb prosthesis users by Gordon Tao, Jayden Singh, Malena Rapaport, Michael Payne and William C Miller in Clinical Rehabilitation
