# Supplementary material for: Predicting walking capacity, walking performance, and life space mobility using socket comfort in lower-limb prosthesis users
Source: Clin Rehabil. 2025 Sep 15;39(12):1673–81. doi: 10.1177/02692155251374927 (PMC12615850; doi:10.1177/02692155251374927)
Supplement: sj-docx-1-cre-10.1177_02692155251374927 - Supplemental material for Predicting walking capacity, walking performance, and life space mobility using socket comfort in lower-limb prosthesis users [file sj-docx-1-cre-10.1177_02692155251374927.docx]

# Supplemental A. All regression Models

## 2-Minute Walk Test

### Final Model

| **Model Summary^b^** | | | | | |  |  |  |  |  |
| --- | --- | --- | --- | --- | --- | --- | --- | --- | --- | --- |
| Model | R | R Square | Adjusted R Square | Std. Error of the Estimate | Durbin-Watson |  |  |  |  |  |
| 1 | .808^a^ | 0.65 | 0.61 | 23.68 | 2.03 |  |  |  |  |  |
| a. Predictors: (Constant), WWT, Age, AmpLevel, SCS, Sex, MonthsSinceAmp, SPPB | | | | | |  |  |  |  |  |
| b. Dependent Variable: T2MWT.meters | | | | | |  |  |  |  |  |
|  |  |  |  |  |  |  |  |  |  |  |
| **ANOVA^a^** | | | | | | |  |  |  |  |
| Model | | Sum of Squares | df | Mean Square | F | Sig. |  |  |  |  |
| 1 | Regression | 66411.45 | 7.00 | 9487.35 | 16.91 | .000^b^ |  |  |  |  |
|  | Residual | 35338.25 | 63.00 | 560.92 |  |  |  |  |  |  |
|  | Total | 101749.70 | 70.00 |  |  |  |  |  |  |  |
| a. Dependent Variable: T2MWT.meters | | | | | | |  |  |  |  |
| b. Predictors: (Constant), WWT, Age, AmpLevel, SCS, Sex, MonthsSinceAmp, SPPB | | | | | | |  |  |  |  |
|  |  |  |  |  |  |  |  |  |  |  |
| **Coefficients^a^** | | | | | | | | | | |
| Model | | Unstandardized Coefficients | | Standardized Coefficients | t | Sig. | 95.0% Confidence Interval for B | | Collinearity Statistics | |
|  |  | B | Std. Error | Beta |  |  | Lower Bound | Upper Bound | Tolerance | VIF |
| 1 | (Constant) | 31.75 | 30.43 |  | 1.04 | 0.30 | -29.07 | 92.56 |  |  |
|  | Age | -0.08 | 0.37 | -0.02 | -0.23 | 0.82 | -0.83 | 0.66 | 0.71 | 1.41 |
|  | Sex | -1.83 | 8.59 | -0.02 | -0.21 | 0.83 | -18.99 | 15.34 | 0.88 | 1.13 |
|  | AmpLevel | -14.70 | 5.32 | -0.21 | -2.76 | 0.01 | -25.33 | -4.07 | 0.92 | 1.08 |
|  | MonthsSinceAmp | 0.01 | 0.01 | 0.05 | 0.57 | 0.57 | -0.02 | 0.03 | 0.77 | 1.30 |
|  | SCS | 6.87 | 2.11 | 0.25 | 3.26 | 0.00 | 2.65 | 11.08 | 0.95 | 1.06 |
|  | SPPB | 3.43 | 0.53 | 0.60 | 6.49 | 0.00 | 2.37 | 4.49 | 0.64 | 1.55 |
|  | WWT | -1.66 | 0.37 | -0.34 | -4.53 | 0.00 | -2.39 | -0.93 | 0.99 | 1.01 |
| a. Dependent Variable: T2MWT.meters | | | | | | | | | | |

### SCS Demographics Only

| **Model Summary^b^** | | | | | |  |  |  |
| --- | --- | --- | --- | --- | --- | --- | --- | --- |
| Model | R | R Square | Adjusted R Square | Std. Error of the Estimate | Durbin-Watson |  |  |  |
| 1 | .525^a^ | 0.28 | 0.22 | 33.53 | 1.91 |  |  |  |
| a. Predictors: (Constant), SCS, Sex, AmpLevel, MonthsSinceAmp, Age | | | | | |  |  |  |
| b. Dependent Variable: T2MWT.meters | | | | | |  |  |  |
|  |  |  |  |  |  |  |  |  |
| **ANOVA^a^** | | | | | | |  |  |
| Model | | Sum of Squares | df | Mean Square | F | Sig. |  |  |
| 1 | Regression | 28175.10 | 5.00 | 5635.02 | 5.01 | .001^b^ |  |  |
|  | Residual | 74200.98 | 66.00 | 1124.26 |  |  |  |  |
|  | Total | 102376.08 | 71.00 |  |  |  |  |  |
| a. Dependent Variable: T2MWT.meters | | | | | | |  |  |
| b. Predictors: (Constant), SCS, Sex, AmpLevel, MonthsSinceAmp, Age | | | | | | |  |  |
|  |  |  |  |  |  |  |  |  |
|  |  |  |  |  |  |  |  |  |
| **Coefficients^a^** | | | | | | | | |
| Model | | Unstandardized Coefficients | | Standardized Coefficients | t | Sig. | 95.0% Confidence Interval for B | |
|  |  | B | Std. Error | Beta |  |  | Lower Bound | Upper Bound |
| 1 | (Constant) | 132.70 | 36.79 |  | 3.61 | 0.00 | 59.24 | 206.15 |
|  | Age | -1.15 | 0.48 | -0.27 | -2.42 | 0.02 | -2.10 | -0.20 |
|  | Sex | 12.71 | 11.78 | 0.12 | 1.08 | 0.28 | -10.80 | 36.23 |
|  | Amp. Level | -20.82 | 7.44 | -0.30 | -2.80 | 0.01 | -35.69 | -5.96 |
|  | Months Since Amp. | 0.04 | 0.02 | 0.24 | 2.23 | 0.03 | 0.00 | 0.07 |
|  | SCS | 7.80 | 2.90 | 0.29 | 2.69 | 0.01 | 2.02 | 13.58 |
| a. Dependent Variable: T2MWT.meters | | | | | | | | |

### SPPB and FSST included

| **Model Summary^b^** | | | | | |  |  |  |  |  |
| --- | --- | --- | --- | --- | --- | --- | --- | --- | --- | --- |
| Model | R | R Square | Adjusted R Square | Std. Error of the Estimate | Durbin-Watson |  |  |  |  |  |
| 1 | .812^a^ | 0.66 | 0.61 | 23.73 | 1.97 |  |  |  |  |  |
| a. Predictors: (Constant), WWT, Age, AmpLevel, SCS, Sex, MonthsSinceAmp, SPPB, FSST.Score | | | | | |  |  |  |  |  |
| b. Dependent Variable: T2MWT.meters | | | | | |  |  |  |  |  |
|  |  |  |  |  |  |  |  |  |  |  |
| **ANOVA^a^** | | | | | | |  |  |  |  |
| Model | | Sum of Squares | df | Mean Square | F | Sig. |  |  |  |  |
| 1 | Regression | 66292.27 | 8.00 | 8286.53 | 14.71 | .000^b^ |  |  |  |  |
|  | Residual | 34362.75 | 61.00 | 563.32 |  |  |  |  |  |  |
|  | Total | 100655.02 | 69.00 |  |  |  |  |  |  |  |
| a. Dependent Variable: T2MWT.meters | | | | | | |  |  |  |  |
| b. Predictors: (Constant), WWT, Age, AmpLevel, SCS, Sex, MonthsSinceAmp, SPPB, FSST.Score | | | | | | |  |  |  |  |
|  |  |  |  |  |  |  |  |  |  |  |
| **Coefficients^a^** | | | | | | | | | | |
| Model | | Unstandardized Coefficients | | Standardized Coefficients | t | Sig. | 95.0% Confidence Interval for B | | Collinearity Statistics | |
|  |  | B | Std. Error | Beta |  |  | Lower Bound | Upper Bound | Tolerance | VIF |
| 1 | (Constant) | 45.01 | 32.29 |  | 1.39 | 0.17 | -19.57 | 109.58 |  |  |
|  | Age | -0.04 | 0.38 | -0.01 | -0.11 | 0.92 | -0.80 | 0.72 | 0.70 | 1.42 |
|  | Sex | -2.76 | 8.64 | -0.03 | -0.32 | 0.75 | -20.03 | 14.52 | 0.88 | 1.14 |
|  | AmpLevel | -13.69 | 5.44 | -0.20 | -2.52 | 0.01 | -24.57 | -2.81 | 0.91 | 1.10 |
|  | MonthsSinceAmp | 0.01 | 0.01 | 0.06 | 0.66 | 0.51 | -0.02 | 0.04 | 0.76 | 1.32 |
|  | SCS | 6.84 | 2.13 | 0.25 | 3.22 | 0.00 | 2.59 | 11.09 | 0.95 | 1.06 |
|  | SPPB | 2.98 | 0.64 | 0.52 | 4.62 | 0.00 | 1.69 | 4.27 | 0.43 | 2.30 |
|  | FSST.Score | -0.78 | 0.62 | -0.15 | -1.26 | 0.21 | -2.01 | 0.46 | 0.40 | 2.52 |
|  | WWT | -1.29 | 0.47 | -0.26 | -2.75 | 0.01 | -2.23 | -0.35 | 0.61 | 1.65 |
| a. Dependent Variable: T2MWT.meters | | | | | | | | | | |

## Step Count

### Final Model

| **Model Summary^b^** | | | | | |  |  |  |  |  |
| --- | --- | --- | --- | --- | --- | --- | --- | --- | --- | --- |
| Model | R | R Square | Adjusted R Square | Std. Error of the Estimate | Durbin-Watson |  |  |  |  |  |
| 1 | .670^a^ | 0.45 | 0.38 | 1155.60 | 1.58 |  |  |  |  |  |
| a. Predictors: (Constant), WWT, SPPB, AmpLevel, SCS, Sex, Age, MonthsSinceAmp | | | | | |  |  |  |  |  |
| b. Dependent Variable: Step.Score | | | | | |  |  |  |  |  |
|  |  |  |  |  |  |  |  |  |  |  |
| **ANOVA^a^** | | | | | | |  |  |  |  |
| Model | | Sum of Squares | df | Mean Square | F | Sig. |  |  |  |  |
| 1 | Regression | 63008561.09 | 7.00 | 9001223.01 | 6.74 | .000^b^ |  |  |  |  |
|  | Residual | 77453985.15 | 58.00 | 1335413.54 |  |  |  |  |  |  |
|  | Total | 140462546.24 | 65.00 |  |  |  |  |  |  |  |
| a. Dependent Variable: Step.Score | | | | | | |  |  |  |  |
| b. Predictors: (Constant), WWT, SPPB, AmpLevel, SCS, Sex, Age, MonthsSinceAmp | | | | | | |  |  |  |  |
|  |  |  |  |  |  |  |  |  |  |  |
| **Coefficients^a^** | | | | | | | | | | |
| Model | | Unstandardized Coefficients | | Standardized Coefficients | t | Sig. | 95.0% Confidence Interval for B | | Collinearity Statistics | |
|  |  | B | Std. Error | Beta |  |  | Lower Bound | Upper Bound | Tolerance | VIF |
| 1 | (Constant) | -969.56 | 1511.88 |  | -0.64 | 0.52 | -3995.91 | 2056.79 |  |  |
|  | Age | 16.61 | 19.00 | 0.10 | 0.87 | 0.39 | -21.42 | 54.64 | 0.79 | 1.27 |
|  | Sex | 1196.06 | 439.70 | 0.28 | 2.72 | 0.01 | 315.90 | 2076.22 | 0.89 | 1.13 |
|  | AmpLevel | -804.36 | 285.29 | -0.29 | -2.82 | 0.01 | -1375.43 | -233.29 | 0.91 | 1.10 |
|  | MonthsSinceAmp | 0.88 | 0.73 | 0.14 | 1.21 | 0.23 | -0.57 | 2.34 | 0.74 | 1.36 |
|  | SCS | 95.29 | 109.52 | 0.09 | 0.87 | 0.39 | -123.94 | 314.51 | 0.92 | 1.09 |
|  | SPPB | 84.77 | 26.81 | 0.38 | 3.16 | 0.00 | 31.09 | 138.44 | 0.64 | 1.56 |
|  | WWT | 1.87 | 18.91 | 0.01 | 0.10 | 0.92 | -35.98 | 39.72 | 0.96 | 1.05 |
| a. Dependent Variable: Step.Score | | | | | | | | | | |

### SCS Demographics Only

| **Model Summary^b^** | | | | | |  |  |  |  |
| --- | --- | --- | --- | --- | --- | --- | --- | --- | --- |
| Model | R | R Square | Adjusted R Square | Std. Error of the Estimate | Durbin-Watson |  |  |  |  |
| 1 | .601^a^ | 0.36 | 0.31 | 1215.89 | 1.53 |  |  |  |  |
| a. Predictors: (Constant), SCS, AmpLevel, Sex, Age, MonthsSinceAmp | | | | | |  |  |  |  |
| b. Dependent Variable: Step.Score | | | | | |  |  |  |  |
| **ANOVA^a^** |  |  |  |  |  |  |  |  |  |
| Model |  | Sum of Squares | df | Mean Square | F | Sig. |  |  |  |
| 1 | Regression | 51009050.10 | 5.00 | 10201810.02 | 6.90 | .000^b^ |  |  |  |
|  | Residual | 90181596.32 | 61.00 | 1478386.82 |  |  |  |  |  |
|  | Total | 141190646.42 | 66.00 |  |  |  |  |  |  |
| a. Dependent Variable: Step.Score | | |  |  |  |  |  |  |  |
| b. Predictors: (Constant), SCS, AmpLevel, Sex, Age, MonthsSinceAmp | | | | |  |  |  |  |  |
|  | | | | | | |  |  |  |
|  |  |  |  |  |  |  |  |  |  |
|  | | | | | | | | | |
| **Coefficients^a^** | | | | | | | | |  |
| Model | | Unstandardized Coefficients | | Standardized Coefficients | t | Sig. | 95.0% Confidence Interval for B | |  |
|  |  | B | Std. Error | Beta |  |  | Lower Bound | Upper Bound |  |
| 1 | (Constant) | 1220.53 | 1399.31 |  | 0.87 | 0.39 | -1577.56 | 4018.62 |  |
|  | Age | -10.67 | 18.51 | -0.06 | -0.58 | 0.57 | -47.69 | 26.35 |  |
|  | Sex | 1460.65 | 451.45 | 0.34 | 3.24 | 0.00 | 557.92 | 2363.37 |  |
|  | Amp. Level | -890.11 | 294.94 | -0.32 | -3.02 | 0.00 | -1479.89 | -300.33 |  |
|  | Months Since Amp. | 1.85 | 0.69 | 0.29 | 2.69 | 0.01 | 0.47 | 3.22 |  |
|  | SCS | 179.69 | 110.66 | 0.17 | 1.62 | 0.11 | -41.58 | 400.97 |  |
| a. Dependent Variable: Step.Score | | | | | | | | |  |

### SPPB and FSST included

| **Model Summary^b^** | | | | | |  |  |  |  |  |
| --- | --- | --- | --- | --- | --- | --- | --- | --- | --- | --- |
| Model | R | R Square | Adjusted R Square | Std. Error of the Estimate | Durbin-Watson |  |  |  |  |  |
| 1 | .665^a^ | 0.44 | 0.36 | 1172.62 | 1.59 |  |  |  |  |  |
| a. Predictors: (Constant), WWT, SPPB, AmpLevel, SCS, Sex, Age, MonthsSinceAmp, FSST.Score | | | | | |  |  |  |  |  |
| b. Dependent Variable: Step.Score | | | | | |  |  |  |  |  |
|  |  |  |  |  |  |  |  |  |  |  |
| **ANOVA^a^** | | | | | | |  |  |  |  |
| Model | | Sum of Squares | df | Mean Square | F | Sig. |  |  |  |  |
| 1 | Regression | 60892556.37 | 8.00 | 7611569.55 | 5.54 | .000^b^ |  |  |  |  |
|  | Residual | 77001777.69 | 56.00 | 1375031.74 |  |  |  |  |  |  |
|  | Total | 137894334.06 | 64.00 |  |  |  |  |  |  |  |
| a. Dependent Variable: Step.Score | | | | | | |  |  |  |  |
| b. Predictors: (Constant), WWT, SPPB, AmpLevel, SCS, Sex, Age, MonthsSinceAmp, FSST.Score | | | | | | |  |  |  |  |
|  |  |  |  |  |  |  |  |  |  |  |
| **Coefficients^a^** | | | | | | | | | | |
| Model | | Unstandardized Coefficients | | Standardized Coefficients | t | Sig. | 95.0% Confidence Interval for B | | Collinearity Statistics | |
|  |  | B | Std. Error | Beta |  |  | Lower Bound | Upper Bound | Tolerance | VIF |
| 1 | (Constant) | -762.00 | 1632.11 |  | -0.47 | 0.64 | -4031.52 | 2507.51 |  |  |
|  | Age | 17.64 | 19.36 | 0.10 | 0.91 | 0.37 | -21.14 | 56.43 | 0.78 | 1.28 |
|  | Sex | 1183.54 | 446.76 | 0.28 | 2.65 | 0.01 | 288.58 | 2078.50 | 0.89 | 1.13 |
|  | AmpLevel | -770.27 | 296.19 | -0.27 | -2.60 | 0.01 | -1363.61 | -176.94 | 0.89 | 1.12 |
|  | MonthsSinceAmp | 0.86 | 0.74 | 0.13 | 1.15 | 0.25 | -0.63 | 2.35 | 0.73 | 1.37 |
|  | SCS | 90.58 | 111.67 | 0.08 | 0.81 | 0.42 | -133.12 | 314.28 | 0.92 | 1.09 |
|  | SPPB | 78.87 | 32.53 | 0.36 | 2.42 | 0.02 | 13.71 | 144.03 | 0.45 | 2.22 |
|  | FSST.Score | -11.60 | 31.07 | -0.06 | -0.37 | 0.71 | -73.85 | 50.64 | 0.43 | 2.35 |
|  | WWT | 7.26 | 24.15 | 0.04 | 0.30 | 0.76 | -41.12 | 55.64 | 0.60 | 1.66 |
| a. Dependent Variable: Step.Score | | | | | | | | | | |

## LSA

### Final Model

| **Model Summary^b^** | | | | | |  |  |  |  |  |
| --- | --- | --- | --- | --- | --- | --- | --- | --- | --- | --- |
| Model | R | R Square | Adjusted R Square | Std. Error of the Estimate | Durbin-Watson |  |  |  |  |  |
| 1 | .453^a^ | 0.21 | 0.12 | 21.59 | 1.66 |  |  |  |  |  |
| a. Predictors: (Constant), WWT, Age, AmpLevel, SCS, Sex, MonthsSinceAmp, FSST.Score | | | | | |  |  |  |  |  |
| b. Dependent Variable: LSA | | | | | |  |  |  |  |  |
|  |  |  |  |  |  |  |  |  |  |  |
| **ANOVA^a^** | | | | | | |  |  |  |  |
| Model | | Sum of Squares | df | Mean Square | F | Sig. |  |  |  |  |
| 1 | Regression | 7459.14 | 7.00 | 1065.59 | 2.29 | .039^b^ |  |  |  |  |
|  | Residual | 28905.52 | 62.00 | 466.22 |  |  |  |  |  |  |
|  | Total | 36364.66 | 69.00 |  |  |  |  |  |  |  |
| a. Dependent Variable: LSA | | | | | | |  |  |  |  |
| b. Predictors: (Constant), WWT, Age, AmpLevel, SCS, Sex, MonthsSinceAmp, FSST.Score | | | | | | |  |  |  |  |
|  |  |  |  |  |  |  |  |  |  |  |
| **Coefficients^a^** | | | | | | | | | | |
| Model | | Unstandardized Coefficients | | Standardized Coefficients | t | Sig. | 95.0% Confidence Interval for B | | Collinearity Statistics | |
|  |  | B | Std. Error | Beta |  |  | Lower Bound | Upper Bound | Tolerance | VIF |
| 1 | (Constant) | 56.51 | 23.98 |  | 2.36 | 0.02 | 8.57 | 104.44 |  |  |
|  | Age | -0.16 | 0.32 | -0.06 | -0.49 | 0.63 | -0.80 | 0.49 | 0.79 | 1.26 |
|  | Sex | -4.40 | 7.76 | -0.07 | -0.57 | 0.57 | -19.91 | 11.10 | 0.90 | 1.11 |
|  | AmpLevel | -3.29 | 4.94 | -0.08 | -0.67 | 0.51 | -13.17 | 6.59 | 0.91 | 1.09 |
|  | MonthsSinceAmp | 0.01 | 0.01 | 0.12 | 0.99 | 0.33 | -0.01 | 0.03 | 0.89 | 1.12 |
|  | SCS | 4.93 | 1.91 | 0.30 | 2.57 | 0.01 | 1.10 | 8.76 | 0.96 | 1.04 |
|  | FSST.Score | -0.87 | 0.46 | -0.28 | -1.88 | 0.06 | -1.79 | 0.05 | 0.59 | 1.71 |
|  | WWT | -0.08 | 0.40 | -0.03 | -0.20 | 0.84 | -0.88 | 0.72 | 0.68 | 1.46 |
| a. Dependent Variable: LSA | | | | | | | | | | |

### SCS Demographics Only

| **Model Summary^b^** | | | | | |  |  |  |  |
| --- | --- | --- | --- | --- | --- | --- | --- | --- | --- |
| Model | R | R Square | Adjusted R Square | Std. Error of the Estimate | Durbin-Watson |  |  |  |  |
| 1 | .286^a^ | 0.08 | 0.01 | 23.05 | 1.50 |  |  |  |  |
| a. Predictors: (Constant), SCS, Sex, AmpLevel, MonthsSinceAmp, Age | | | | | |  |  |  |  |
| b. Dependent Variable: LSA | | | | | |  |  |  |  |
| **ANOVA^a^** |  |  |  |  |  |  |  |  |  |
| Model |  | Sum of Squares | df | Mean Square | F | Sig. |  |  |  |
| 1 | Regression | 3130.09 | 5.00 | 626.02 | 1.18 | .329^b^ |  |  |  |
|  | Residual | 35071.32 | 66.00 | 531.38 |  |  |  |  |  |
|  | Total | 38201.41 | 71.00 |  |  |  |  |  |  |
| a. Dependent Variable: LSA | |  |  |  |  |  |  |  |  |
| b. Predictors: (Constant), SCS, Sex, AmpLevel, MonthsSinceAmp, Age | | | | | | |  |  |  |
|  | | | | | | |  |  |  |
|  |  |  |  |  |  |  |  |  |  |
|  | | | | | | | | | |
| **Coefficients^a^** | | | | | | | | |  |
| Model | | Unstandardized Coefficients | | Standardized Coefficients | t | Sig. | 95.0% Confidence Interval for B | |  |
|  |  | B | Std. Error | Beta |  |  | Lower Bound | Upper Bound |  |
| 1 | (Constant) | 57.57 | 25.29 |  | 2.28 | 0.03 | 7.07 | 108.06 |  |
|  | Age | -0.23 | 0.33 | -0.09 | -0.72 | 0.48 | -0.89 | 0.42 |  |
|  | Sex | -1.29 | 8.10 | -0.02 | -0.16 | 0.87 | -17.45 | 14.87 |  |
|  | Amp. Level | -4.53 | 5.12 | -0.11 | -0.88 | 0.38 | -14.75 | 5.69 |  |
|  | Months Since Amp. | 0.01 | 0.01 | 0.12 | 0.95 | 0.35 | -0.01 | 0.04 |  |
|  | SCS | 3.96 | 1.99 | 0.24 | 1.99 | 0.05 | -0.01 | 7.93 |  |
| a. Dependent Variable: LSA | | | | | | | | |  |

### SPPB and FSST included

| **Model Summary^b^** | | | | | | |  |  |  |  |  |
| --- | --- | --- | --- | --- | --- | --- | --- | --- | --- | --- | --- |
| Model | R | R Square | Adjusted R Square | | Std. Error of the Estimate | Durbin-Watson |  |  |  |  |  |
| 1 | .453^a^ | 0.21 | 0.10 | | 21.77 | 1.66 |  |  |  |  |  |
| a. Predictors: (Constant), WWT, Age, AmpLevel, SCS, Sex, MonthsSinceAmp, SPPB, FSST.Score | | | | | | |  |  |  |  |  |
| b. Dependent Variable: LSA | | | | | | |  |  |  |  |  |
|  |  |  |  | |  |  |  |  |  |  |  |
| **ANOVA^a^** | | | | | | | |  |  |  |  |
| Model | | Sum of Squares | df | | Mean Square | F | Sig. |  |  |  |  |
| 1 | Regression | 7459.83 | 8.00 | | 932.48 | 1.97 | .066^b^ |  |  |  |  |
|  | Residual | 28904.83 | 61.00 | | 473.85 |  |  |  |  |  |  |
|  | Total | 36364.66 | 69.00 | |  |  |  |  |  |  |  |
| a. Dependent Variable: LSA | | | | | | | |  |  |  |  |
| b. Predictors: (Constant), WWT, Age, AmpLevel, SCS, Sex, MonthsSinceAmp, SPPB, FSST.Score | | | | | | | |  |  |  |  |
|  |  |  |  | |  |  |  |  |  |  |  |
| **Coefficients^a^** | | | | | | | | | | | |
| Model | | Unstandardized Coefficients | | Standardized Coefficients | | t | Sig. | 95.0% Confidence Interval for B | | Collinearity Statistics | |
|  |  | B | Std. Error | | Beta |  |  | Lower Bound | Upper Bound | Tolerance | VIF |
| 1 | (Constant) | 57.16 | 29.62 | |  | 1.93 | 0.06 | -2.07 | 116.38 |  |  |
|  | Age | -0.16 | 0.35 | | -0.06 | -0.47 | 0.64 | -0.85 | 0.53 | 0.70 | 1.42 |
|  | Sex | -4.35 | 7.92 | | -0.07 | -0.55 | 0.58 | -20.20 | 11.49 | 0.88 | 1.14 |
|  | AmpLevel | -3.30 | 4.99 | | -0.08 | -0.66 | 0.51 | -13.28 | 6.67 | 0.91 | 1.10 |
|  | MonthsSinceAmp | 0.01 | 0.01 | | 0.12 | 0.92 | 0.36 | -0.01 | 0.04 | 0.76 | 1.32 |
|  | SCS | 4.94 | 1.95 | | 0.30 | 2.53 | 0.01 | 1.04 | 8.84 | 0.95 | 1.06 |
|  | SPPB | -0.02 | 0.59 | | -0.01 | -0.04 | 0.97 | -1.21 | 1.16 | 0.43 | 2.30 |
|  | FSST.Score | -0.88 | 0.57 | | -0.28 | -1.56 | 0.12 | -2.01 | 0.25 | 0.40 | 2.52 |
|  | WWT | -0.07 | 0.43 | | -0.03 | -0.17 | 0.86 | -0.93 | 0.79 | 0.61 | 1.65 |
| a. Dependent Variable: LSA | | | | | | | | | | | |
